# Evaluation of the Effectiveness of Esterified Hyaluronic Acid Fibers on Bone Regeneration in Rat Calvarial Defects

**DOI:** 10.1155/2018/3874131

**Published:** 2018-06-28

**Authors:** Omer B. Agrali, Selin Yildirim, Hafize O. Ozener, Kemal N. Köse, Dilek Ozbeyli, Merva Soluk-Tekkesin, Leyla Kuru

**Affiliations:** ^1^Department of Periodontology, Faculty of Dentistry, Marmara University, Istanbul, Turkey; ^2^Department of Physiology, Faculty of Medicine, Marmara University, Istanbul, Turkey; ^3^Department of Tumor Pathology, Institute of Oncology, Istanbul University, Istanbul, Turkey

## Abstract

Hyaluronic acid (HA) constitutes one of the major components of the extracellular matrix domain in almost all mammals. The aim of this study was to evaluate the regenerative capacity of HA matrix in rat calvarial bone defects and compare with those of different combinations of resorbable collagen membrane (M) and bovine-derived xenograft (G). Twenty-four 3-month-old male Sprague-Dawley rats weighing 200-250 g were included. Control group was created by leaving one defect empty from 2 critical size defects with 5 mm diameter formed in the calvarial bones of 8 rats. In the same rats, the other defect was treated with HA matrix alone. One of the 2 defects formed in other 8 rats was treated with HA+G and the other with HA+M. One of the 2 defects formed in the remaining 8 rats was treated with G+M and the other with HA+G+M. The animals were sacrificed at 4 weeks. Histologic, histomorphometric, and immunohistochemical analyses were performed. Both HA matrix alone and its combinations with G and M supported new bone formation (NBF). However, NBF was significantly greater in G+M and HA+G+M groups compared to control and HA alone (P<0.001). Bone morphogenetic protein-2 was expressed with varying degrees in all groups, without any difference among them. Within the limitations of the present study, HA matrix, used alone or in combination with G and M, did not contribute significantly to bone regeneration in rat calvarial bone defects.

## 1. Introduction

In recent years, the extent of the use of degradable materials like hyaluronic acid (HA) for the reconstruction of soft and hard tissue deformities has been mostly increased in dental field. HA, a glycosaminoglycan structured biomolecule, is a major component of the extracellular matrix in almost all mammals. It was first discovered and isolated from the cow eye by Karl Meyer and John Palmer in 1934 [1]. Since then, the use of HA-based biomaterials with the aim of influencing and enhancing the wound healing manner has been stated to be effective especially in the regenerative procedures [2–4]. Participation in numerous essential biological events including cell adhesion, proliferation, differentiation, and cellular signaling makes HA attractive for oral applications [5]. From the therapeutic point of view, HA-based products are available for the treatment of oral ulcer [6], gingivitis [7], bone defects [8], and periodontal defects [9].

Although current developments in biomaterial science bring forward some alternatives, they are still far from achieving exact proper solutions. The limitations of biomaterials may represent some structural and functional properties including viscosity, elasticity, biodegradability, molecular weight, and concentration leading biological actions which signify essential meanings for tissue formation. Recently, novel methods have been proposed especially in hydrogel structured scaffold construction to produce original efficient HA-based biomaterials with improved mechanical and morphological properties [10]. Some of these semi-synthetic materials are obtained by conducting a chemical revision based on the esterification of carboxyl groups in the purified HA molecule which can be organized by chemical agents leading to enhance the biological efficacy of HA [10]. Esterified HA has favorable biologic abilities in bone tissue regeneration observed in the existence of chondrocytes and mesenchymal stem cells [11–13].

Clinical studies evaluating the regenerative response of a biopolymer matrix composed of fibers in esterified HA structure suggest promising results in terms of attachment gain and bone formation rate [8, 9, 14–16]. Due to the lack of substantial clinical evidence, heterogeneity of study results, and uncertainties in effect size of HA and application method, the use of esterified HA fibers could not be acclaimed in the regenerative periodontal therapies [5]. Therefore, its efficiency is still needed to be clarified.

Guided bone regeneration (GBR) is defined as a procedure providing a selective barrier placed between the bony defect site and the mucoperiosteal flap to prevent the repopulation of rapidly proliferating epithelial and gingival connective tissue cells from the defect area [17]. Thus, osteoblast cells which are relatively slow-growing cells capable of forming bone will selectively proliferate in the defect site and thereby bone regeneration will occur [17]. In several studies, it has been shown that different types of barrier membrane prevent the migration of undesired cells into the wound area and at the same time permit the migration of regenerative cells within the confinement area [18–21]. GBR is often combined with bone grafting procedures [17, 22–25]. Bovine-derived xenograft (G) is commonly used in orthopedics, neurosurgery, oral maxillofacial surgery, and periodontology fields for purposes such as providing bone support, skeletal defect repair, and socket preservation [26].

Bone morphogenetic protein (BMP), a member of transforming growth factor-*β* family, is synthesized by osteoblasts and osteocytes and primarily located in bone and dentin in adult mammals [27]. The presence of BMP has been shown to stimulate migration and conversion of mesenchymal cells into osteoblasts, storage of the bone matrix, and mineralization of the newly stored bone matrix [27]. BMP-2 is the most abundant osteoinductive protein among BMPs [27].

Current evidence on the use of HA-containing biomaterials in GBR is inadequate. Therefore, the present study aimed to evaluate the bone regenerative effect of HA matrix alone and compare in combination with G and resorbable collagen membrane (M) by assessing the new bone formation and BMP-2 expression in critical size rat calvarial bone defects investigating the null hypothesis that the use of esterified HA fibers would not affect the healing process.

## 2. Materials and Methods

### 2.1. Animals

Twenty-four, 3-month-old, male Sprague-Dawley rats weighing 200-250 g were included to the study. This animal study was conducted in the Marmara University Experimental Animal Research Laboratory approved by Marmara University Animal Experiments Ethics Committee (protocol no=08.2015.mar). Throughout the experimental period, animals were maintained under a controlled light and dark cycle 12:12 h, light on at 8:00 am at an ambient 21±2°C temperature, and fed with standard laboratory pellet food. Drinking water was available* ad libitum*.

According to the power analysis performed by using the values (0.38 mm^2^ mean new bone formation, 0.158 mm^2^ standard deviation with 95% power, and significance was set at p<0.05 level) obtained from an animal study [28] having similar defect size and follow-up period with our study and comparing new bone formation between the groups, it was calculated that a minimum of 5 defects should be included into each group. Eight defects were decided to be included into each group considering the possible loss of the animals for any reason.

### 2.2. Surgical Procedures

**Figure 1:**
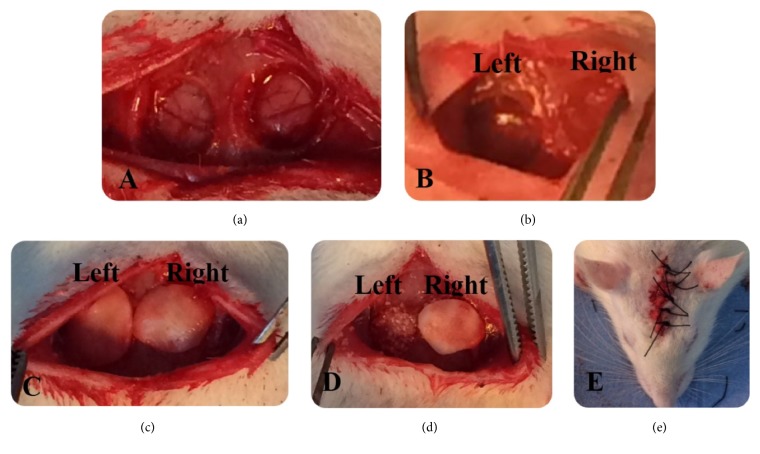
Representative experimental figures of the groups. (a) Critical size calvarial defects, (b) control group (Left) and HA group (Right), (c) G+M (Left) and HA+M (Right) groups, (d) HA+G (Left) and HA+G+M (Right) groups, and (e) suturing.

Intramuscular injection of 3 mg/kg xylazine hydrochloride (Rompun, Bayer, Leverkusen, Germany) and 35 mg/kg ketamine hydrochloride (10% Ketasol, Richter Pharma AG, Wels, Austria) was applied for general anesthesia of the rats. Under sterile conditions, the dorsal part of the cranium was shaved and incised through the skin, muscles, and periosteum to expose the calvarium. Two critical size defects with 5 mm diameter were created in the right and left sides of the parietal bone without causing any injury to the underlying dura mater (Figure 1(a)). Under constant sterile saline irrigation, a trephine bur inserted into a low speed handpiece was used to create the calvarial defects. A total of 48 defects were divided into 6 groups as follows: control group (n=8): defects were left empty; HA group (n=8): defects were filled with HA alone (HYALOSS™ matrix, Fidia Advanced Biopolymers, Abano Terme, Italy) (Figure 1(b)); G+M group (n=8): defects were filled with G (Bio-Oss, Geistlich Pharma AG, Wolhusen, Switzerland) and covered with M (BioGide, Geistlich Pharma AG, Wolhusen, Switzerland); HA+M group (n=8): defects were filled with HA and covered with M (Figure 1(c)); HA+G group (n=8): defects were filled with HA and G; G+HA+M group (n=8): defects were filled with HA and G and then covered with M (Figure 1(d)). The skin was primarily sutured with nonresorbable 3/0 silk sutures (Doğsan, Trabzon, Turkey) (Figure 1(e)). Postoperative infection control was provided by using intramuscular injection of 25 mg/kg antibiotic Ceftriaxone (Rocephin, Roche, Nutley, New Jersey, USA) for 3 days and 4 mg/kg analgesic Carprofen (Rimadyl, Pfizer, New York, USA) 24 h a day for 3 days, starting immediately after the operation. Sutures were removed at day 7 postoperatively. The animals were euthanized by anesthetic overdose and sacrificed at 4 weeks.

### 2.3. Histologic and Histomorphometric Evaluation

The skulls were fixed in 10% buffered formalin for 1 week. Then, they were decalcified in 10% formic acid+10% sodium citrate solution for 1 month. Paraffin blocks prepared from routinely processed decalcified specimens were cut into 4 *μ*m slices followed by staining with hematoxylin and eosin. The sections were evaluated histologically under a light microscope (Olympus BX60; Olympus Optical Co. Ltd., Japan) attached to a color video camera and connected to a computer for the presence of inflammatory change and foreign body reaction. The infection, necrosis, and foreign body reaction were evaluated as ‘-' if absent and ‘+' if present.

The defect regions were captured using the camera and displayed on the computer monitor for histomorphometric measurements in order to evaluate the amounts of new bone formation and residual bone graft. All measurements were performed with Image Analysis Software (Olympus® Image Analysis Software 5.0, Tokyo, Japan) by a blinded pathologist (MST) at three different evaluation times with 1-week interval. The mean value revealed from these three evaluations was assigned as the value which was used for statistical analysis.

The sections were evaluated at x40, x100, and x200 magnifications for new bone formation and residual graft which were calculated in 1 or 2 contiguous and consecutive microscopic fields, depending on the size of related microscopic area. The proportions of the area occupied by newly formed bone and residual grafts were measured and confined to a total area.

### 2.4. Immunohistochemical Staining and Evaluation

The sections were deparaffinized and antigen retrieval was performed for immunohistochemistry. After microwave incubation of the peroxyblock, followed by the ultra V block procedure, primary antibody against BMP-2 (Polyclonal antibody, ENT0498, Elabscience Biotechnology Co., Ltd., Houston, USA) was applied. Following this process, biotinylated secondary antibody (HRP conjugated polyclonal antibody, Sc-2030, Lot # D1504, Santa Cruz Biotechnology, Texas, USA), streptavidin peroxidase and substrate-chromogen solution were applied. Nuclear counterstaining was done with hematoxylin. The sections were evaluated under the light microscope and the score was made up of 0-5% positive cells as (-), 5-30% positive cells as (+), 30-60% positive cells as (++), and 60% and more positive cells as (+++).

### 2.5. Statistical Analysis

Analyses of data were performed by using a commercially available statistical software (SPSS® 15.0 for Windows, Chicago, IL, USA). For each group, amounts of newly formed bone and residual graft material were separately calculated by using mean values and standard deviations. Nonparametric tests were conducted because Kolmogorov-Smirnov test showed that data were not normally distributed. Intergroup comparisons were made by Kruskal-Wallis test followed by Mann–Whitney U test with post hoc Bonferroni correction for paired comparisons. Immunohistochemical data were analyzed by using Chi-Square test. Statistical significance was set at p<0.05 level.

## 3. Results

There was no animal loss throughout the study period. Uneventful healing was achieved in all animals without any postoperative complication. New bone formation was observed in all groups. Moreover, the amount of newly formed bone in the groups G+M and HA+G+M was significantly greater than that in the control and HA groups (p<0.001, p<0.01) (Table 1). In the G+M, HA+G, and HA+G+M groups, where G was applied, residual graft material was detectable. However, amounts of the residual graft material were similar in all groups (p>0.05) (Table 1).

**Figure 2:**
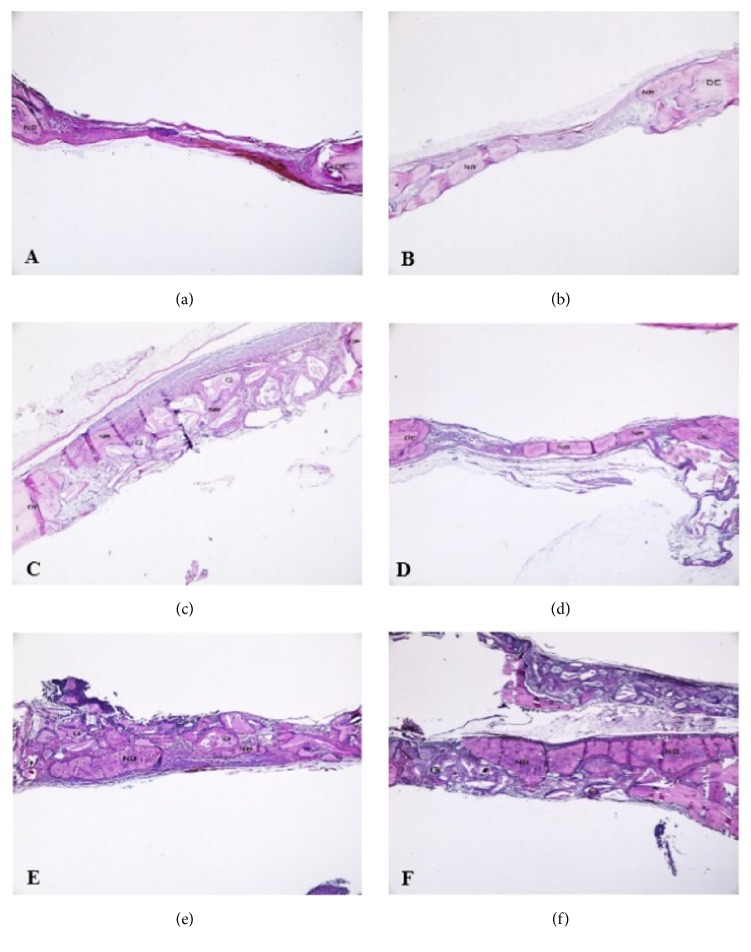
Representative histological sections of the groups. (a) Control group, (b) HA group, (c) G+M group, (d) HA+M group, (e) HA+G group, and (f) HA+G+M group (all figures H&E x40, NB: new bone, G: graft, and DE: defect edge).

The representative histologic sections of the groups are shown in Figure 2. No histopathological damage of dura mater was observed in any of the specimens after the creation of calvarial defects. In all groups, the calvarial defects were not completely filled with regenerated bone. Newly formed bone tissue surrounded by osteoblasts was limited to areas close to the borders of the surgically created defects in all specimens. All groups exhibited increased osteoblastic activity (Figure 2).

**Figure 3:**
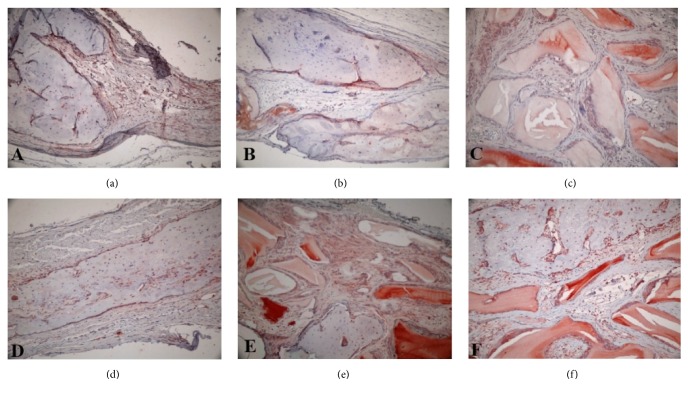
Demonstrative pictures of BMP-2 immunohistochemical staining. (a) Control group, (b) HA group, (c) G+M group, (d) HA+M group, (e) HA+G group, and (f) HA+G+M group (all figures BMP-2x200).

Evaluation of immunohistochemical staining revealed that all groups showed BMP-2 positivity in varying degrees (Figure 3); however no statistically significant difference was found among them (p>0.05) (data not shown). The distinct staining was observed at osteoblasts close to newly formed bone. Mesenchymal tissue cells and defect edges expressed also positive staining. Even though there were no statistically differences (p>0.05), the staining in the G+M, HA+G, and HA+G+M groups containing graft material was observed stronger than the control and HA groups without graft material. The BMP-2 positive cells in the mesenchymal tissue were considered as the precursors of osteoblasts. Some osteocytes gave also positive reaction to the BMP-2 antibody.

## 4. Discussion

The biological activity, boundaries, and manner of the application of HA material, which is widely used in dermatology as far as medical dentistry, have not yet been clearly established. Furthermore, there is a lack of knowledge on the application mode of HA matrix either alone or combined with G and/or M. Our research is the first animal study investigating systematically and comparatively the limitations and effectiveness of HA-containing bioactive matrix alone and in combination. Our results exerted that no additional improvement could be achieved on rat calvarial bone regeneration by the use of esterified HA fibers.

In this study, the defect model of critical size in rat calvaria was used to investigate the effectiveness of HA-containing biomaterial on bone regeneration. Generally, the critical size of the rat calvarial bone defect model includes only one defect with 8 mm diameter. However, it is possible to use defects with lower size in order to be able to create 2 defects in one rat allowing use of relatively small number of animals. Therefore, in our study, two calvarial defects of 5-mm in size were created in one rat similar to the studies reviewed by Muschler et al. [29]

As a bone graft, G is obtained by removing the natural bone mineral after it has been separated from the organic components by standing for 24 h with ethylenediamine [30]. Due to its porous structure and high mineral component, G integrates into the existing bone by providing an osteoconductive scaffold [30]. Kohal et al. [31] reported the finding that G contributes higher values of new bone formation when used in combination with resorbable collagen barrier membranes. This evidence was supported with systematic review articles [21, 22]. In this study, it is assumed that the bioactive matrix containing HA material, which has been shown to have positive effects on wound healing, will positively contribute to the results obtained with the combination of G and M used for bone regeneration in the concept of GBR. However, our results did not support this hypothesis. On the other hand, the results obtained from G and M combination were in line with the literature [21, 22].

The HA activity is mostly associated with the molecular weight and concentration of HA acting in the biomaterial [5]. Results which are reviewed from the studies investigating* in vitro* and* in vivo* efficacy of the HA molecule applied at varying concentrations and molecular weights are inconsistent [5]. Some researchers reported enhanced cell differentiation by high molecular weight-HA [4, 32]; some others showed no effect of high molecular weight-HA on cell differentiation [33, 34]. While low molecular weight-HA has been stated to stimulate angiogenesis [35] and possess inflammatory effects [36], high molecular-HA has been reported to inhibit angiogenesis [37] and have anti-inflammatory effects [38, 39]. Zhao et al. [5] reviewed* in vivo* bone regenerative effect of HA with varying molecular weight and concentration. It has been reported that HA with molecular weight of 35 kDa to 6000 kDa and concentration of 10 mg/ml to 26 mg/ml can improve new bone formation [5]. The HA-based biomaterial used in our study contains HA at a molecular weight range of 180-200 kDa and at a concentration range of 20-60 mg/ml [14, 40].

Tissue regeneration involves complex, early, and late healing manners including adhesion, proliferation, differentiation, and functioning of the cells [5]. Adhesion and proliferation activities of the cells which mainly take place in the early wound healing period differ under HA application [5]. Takeda et al. [34] observed greater cell adhesion and proliferation but no cell differentiation effects suggesting the HA related events at early healing phase more willingly than those at late phase. On the other hand, no cell proliferative effect was detected with HA application compared with that of cells not treated by HA [33]. Different cellular responses of HA defining its early healing effects are still needed to be clarified.

The main component of our HA-based test material was successfully used in cases of synthetic bioskin and biosynthetic osteocartilage reconstruction [41–44]. In* in vivo* animal studies in which the effects of HA-containing biomaterials on bone regeneration were evaluated, HA was used generally as a carrier and was shown to increase bone regeneration [2, 45–48]. For example, thiol-modified HA combined with polyethylene glycol provided a sustained release of BMP-2, resulting in ectopic bone formation in the hind limbs of the rats [47]. Bone formation was observed by subperiosteal administration of the injectable HA in a minimally invasive manner in the rat calvarial region [2]. It has also been shown that growth and differentiation factor 5 or simvastatin carried out by HA significantly increased osteogenesis [45, 46]. Glycidyl methacrylate-modified HA hydrogels were administered alone or in combination with BMP-2 into rat calvarial bone defects and higher level of mineralization was detected in the group treated with BMP-2 than the control group [48]. HA with vascular endothelial growth factor or BMP-2 has been shown to increase healing in rat calvarial bone defects [48]. In an animal study evaluating the healing of alveolar sockets after tooth extraction, one of the defects was left empty and another was treated with HA, and the results demonstrated that the detected amount of BMP-2 was greater and trabecular formation was faster in the HA-treated group [49]. On the other hand, in the sheep femur defect model, injectable HA was unable to produce statistically significant bone formation when administered alone or in combination with BMP-2 [50]. In a dog model, tricalcium phosphate was used alone or in combination with HA in the treatment of bony defects created in the radii and no significant difference in the new bone formation was found between the groups [51]. No significant contribution was obtained in terms of new bone formation and BMP-2 expression after application of HA-based bioactive matrix alone or combined with G and/or M in rat calvarial bone defects at the end of the 4-week healing period.

As a limitation of this study, it can be asserted that there is a need for studies involving a longer follow-up period with an evaluation performed at different time points in which the efficacy of this HA-based biomaterial is assessed in different tissues and animal models also in noncritical defects.

## 5. Conclusions

Within the limits of this study, our findings demonstrated that the application to rat calvarial bone defects of HA-based bioactive matrix, alone or in combination with G and M, had no additional effect on bone regeneration after 4 weeks of healing.

## Figures and Tables

**Table 1:** Comparison of histomorphometric parameters among the groups.

	**Groups**						**P** ^a^
	**Control**	**HA**	**G+M**	**HA+M**	**HA+G**	**HA+G+M**	
	**N=8**	**N=8**	**N=8**	**N=8**	**N=8**	**N=8**	
	**Mean±SD**	**Mean±SD**	**Mean±SD**	**Mean±SD**	**Mean±SD**	**Mean±SD**	
**New bone formation (mm** ^**2**^ **)**	0.15±0.14	0.12±0.13	0.79±0.43^b,c^	0.35±0.35	0.38±0.20	0.73±0.30^b,c^	0.000

**Residual graft (mm** ^**2**^ **)**	-	-	0.63±0.21	-	0.48±0.16	0.63±0.16	0.183

^a^ *Kruskal Wallis*, P<0.05.

^b^P<0.001 different from the control group, Mann–Whitney U-test with post hoc Bonferroni correction.

^c^P<0.01 different from the HA group, Mann–Whitney U-test with post hoc Bonferroni correction.

## Data Availability

The data used in our study can be shared with interested parties if requested.
